# DFT Computed Dielectric Response and THz Spectra of Organic Co-Crystals and Their Constituent Components

**DOI:** 10.3390/molecules24050959

**Published:** 2019-03-08

**Authors:** Joseph W. Bennett, Michaella E. Raglione, Shalisa M. Oburn, Leonard R. MacGillivray, Mark A. Arnold, Sara E. Mason

**Affiliations:** Department of Chemistry, University of Iowa, Iowa City, IA 52242, USA; joseph-bennett@uiowa.edu (J.W.B.); michaella-raglione@uiowa.edu (M.E.R.); shalisa-oburn@uiowa.edu (S.M.O.); len-macgillivray@uiowa.edu (L.R.M.); mark-arnold@uiowa.edu (M.A.A.)

**Keywords:** DFT-D, co-crystals, crystal packing, dispersion, dielectric response

## Abstract

Terahertz (THz) spectroscopy has been put forth as a non-contact, analytical probe to characterize the intermolecular interactions of biologically active molecules, specifically as a way to understand, better develop, and use active pharmaceutical ingredients. An obstacle towards fully utilizing this technique as a probe is the need to couple features in the THz regions to specific vibrational modes and interactions. One solution is to use density functional theory (DFT) methods to assign specific vibrational modes to signals in the THz region, coupling atomistic insights to spectral features. Here, we use open source planewave DFT packages that employ ultrasoft pseudopotentials to assess the infrared (IR) response of organic compounds and complex co-crystal formulations in the solid state, with and without dispersion corrections. We compare our DFT computed lattice parameters and vibrational modes to experiment and comment on how to improve the agreement between theory and modeling to allow for THz spectroscopy to be used as an analytical probe in complex biologically relevant systems.

## 1. Introduction

The medical industry has begun to utilize active pharmaceutical ingredients (APIs) in a co-crystalline form to enhance the functionalities of their products [1,2,3]. These solids are unique, as they contain two non-ionic components, an API and a coformer. The API and coformer can bind through van der Waals forces and non-covalent interactions such as hydrogen bonding, similar to biologics such as DNA and proteins [4,5]. Binding through non-ionic forces can enable more flexibility in crystalline engineering, removing the need to form an ionic bond. Understanding the intermolecular forces holding these co-crystals together can help to better understand, predict and control their physical properties. The creation of co-crystal APIs is a strategy that has shown to influence solubility and bioavailability [6], thermal stability [7], and has also found use in the control of product concentrations in the development of new types of therapeutics [8].

To better understand the structures of co-crystals with APIs, researchers have utilized terahertz (THz) spectroscopy and low frequency Raman spectroscopy (LFRS). Both LFRS and THz spectroscopy probe frequencies between 3–300 cm${}^{-1}$, where the prominent vibrational modes are related to the packing of the constituent organics in a crystal and the intermolecular forces that hold them in place [9,10]. These spectroscopic techniques have already proven useful for identifying different polymorphs that form when mixing multiple organic compounds [10,11,12], for the in situ monitoring of crystallization and structural transformations [13,14], as well as uncovering solid state transition mechanisms [15,16]. THz spectroscopy is potentially a powerful tool in probing biological systems, because it is compatible with online measurements, remote sampling, and three-dimensional imaging [17], although little work has been published on large complex systems beyond binary and tertiary co-crystals. Most THz research has focused on smaller organic-based systems (≈50–150 atoms/unit cell) where several groups have worked towards using theory and modeling to assign the vibrational modes found in both THz absorption and low frequency Raman spectroscopies [10,18,19,20].

An open question in the theory and modeling of materials is: “How closely can calculated properties match experimentally determined information?” This is especially difficult to answer when computing the measurable properties of a complex material with multiple components and potentially nonlinear behavior, or if changes in the chemical environment, such as the formation of hydrogen bonds or solvation, must be taken into account. The atomistic detail needed to address these issues can be gleaned from using methods based on quantum mechanics, specifically density functional theory (DFT). DFT methods have been around since the mid-1960s [21,22] and allowed for calculation of properties in the solid state for simple systems, such as the ordering and electronic structure of Si [23] and ZnS semiconductors [24]. Since the 1990s improvements in pseudopotential design and construction [25,26,27,28,29,30] and exchange-correlation functionals [31,32,33], as well as the distribution of open source software packages [34,35] and the advent of highly parallelized computing have placed DFT methods at the forefront of accurate and reliable methods based in quantum mechanics.

How organic molecules pack into crystal structures is ultimately governed by intermolecular interaction patterns. While chemists can qualitatively identify systems in which, for example, π–π stacking, hydrogen bonding, and long-range dispersion forces occur, it is extremely difficult to predict from such an analysis what crystal structure will be preferred for a given organic molecule, either individually or with a co-former. Here, first-principles modeling based on DFT calculations becomes attractive as a method known to be reliable for modeling the structure and properties of inorganic crystal structure materials [36,37,38,39,40,41].

DFT calculations are upheld by materials chemistry as the gold-standard in quantum mechanical modeling owing to desirable balance between computational speed and accuracy. However, directly applying these methods to organic crystalline materials is complicated by the differing intermolecular forces active in these materials, noted above. While DFT is in principle an exact method, in practice the local density approximation, LDA (local density approximation) (and extensions to it, such as the generalized-gradient approximation, GGA) to the true, universal exchange-correlation functional are employed. In effect, this means that, while all non-relativistic electronic effects are included in the formal exact DFT functional, including van der Waals interactions, the long-range, non-local correlations that contribute to van der Waals forces are not accounted for in calculations that use LDA or GGA functionals. Solutions to this problem are complicated, as local effects are, by construction, included. Therefore, extending DFT approximations to include long-range forces is not as simple as just adding additional terms. Indeed, while such efforts to extend DFT came to the forefront in the early-2000s, the methods themselves [42,43,44,45,46,47], as well as best-practices for their applications [48,49], are still active areas of research.

Of the various approaches to extend DFT to include long-range electron correlation interactions, D2 (and more recently D3) dispersion coefficient methods, [43,46,50], referred to as DFT-D, are popular based on the relatively simple mathematical form, improved predictions, and use of adjustable parameters. While it is beyond the scope of this manuscript to review the methods (those this has been done elsewhere, for example, Ref. [51]), it can be stated briefly that DFT-D empirical parameters are determined by fits done to conformational and interaction energies computed using high-level quantum mechanical calculations. While in some implementations the fit parameters are adjustable, this is not universally the case.

Here, we present calculations that use open source DFT packages (that employ pseudopotentials and a planewave basis set and include dispersion corrections) to obtain the lattice parameters and vibrational modes of organic co-crystals and their constituent components. It is impossible to know which empirically tuned variant of DFT-D will offer the best performance for the organic co-crystals under study here. Given the lack of benchmarking information in the literature, we elect to test the performance of Grimme-D2 DFT-D calculations, compared to standard DFT-GGA. Our choice of Grimme-D2 DFT-D for this study is motivated by the fact that, in some implementations, the empirical parameter controlling the dispersion corrections is tunable by the user, enabling us to explore how its variation affects predictions of the bulk lattice constants. Future studies could go on to test other DFT-D functionals, and this would contribute to the developing understanding of which correction schemes offer optimal performance for organic crystals. We compare our DFT calculated results, both with and without dispersion corrections, to experimentally determined lattice constants and absorptions over THz frequencies. We use the information obtained with DFT to analyze the dielectric response of the systems studied here in order to elucidate trends in the types of functional groups present, crystal packing, and ultimately how one could use dielectric response as a tailorable property when designing and understanding complex organic co-crystal API formulations. We focus on identifying infrared (IR) active modes, their contributions to the overall dielectric response, and how closely they match the IR active modes measured in THz absorption spectroscopy. We use the work presented here to establish a baseline for future studies where recently developed methods to model organic solids could be used to improve upon the disagreements between theory and experimental endeavors.

## 2. Results and Discussion

**Figure 1:**
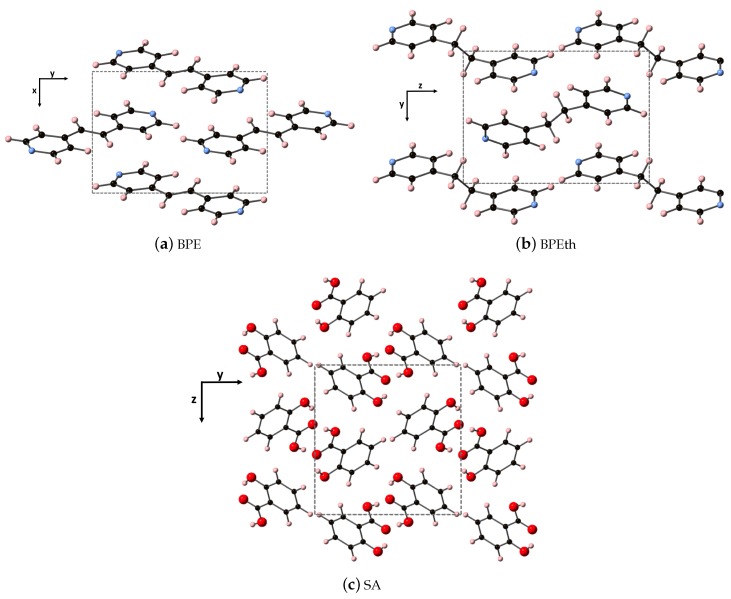
Shown here are the co-crystal components (**a**) BPE, (**b**) BPEth, and (**c**) SA. Atoms of carbon, hydrogen, nitrogen, and oxygen are depicted as black, light pink, light blue, and red spheres, respectively. Dashed lines represent the crystallographic axes of the primitive lattice unit cell for each of the components.

The fully relaxed monoclinic crystal structures of the components used to create the co-crystals (discussed in the next subsection) are depicted in Figure 1. All three are indexed in space group 14, where all three have monoclinic angle β > 90${}^{\circ }$. Shown are the repeat units of 1,2-bis(4-pyridyl)ethylene (BPE), 1,2-bis(4-pyridyl)ethane (BPEth), and salicylic acid (SA) where the direction containing the greatest dispersion is marked by the vertical axis. The coformers BPE and BPEth differ in the functional group connecting the planar C${}_{5}$N ring units; inspection of Figure 1a,b shows that BPE contains an alkene C${}_{2}$H${}_{2}$ unit and BPEth contains an alkane C${}_{2}$H${}_{4}$ unit, and that this influences the relative orientation of packing of these structures. In neither case is there a significant number of intermolecular (primary) hydrogen bonds, and a consequence of this is packing directions in which dispersion is significant. Table 1 contains the computed crystallographic parameters of the fully relaxed systems. *a*, *b*, and *c* are the lattice constants of the primitive unit cell, and β is the angle between *a* and *c*.

Inspection of Table 1 shows that DFT without dispersion corrections overestimated BPE lattice constants *a* and *c* by ≈ 40 and 20 %, and BPEth lattice constant *b* by ≈ 20%, relative to experiments. Inclusion of dispersion (DFT-D) reduces these errors to ≈−7 and −3 %, and ≈−4 %, for BPE and BPEth, respectively. Another error that is not typically reported in DFT studies is that of β, the angle between *a* and *c*. For the monoclinic BPE unit cell, DFT overestimates this angle by over 25%, and this is because the errors in *a* and *c* are so large. DFT-D decreases the error in β to ≈−2.5%. The experimentally determined lattice parameters for BPE [52], BPEth [53], and SA [54] are given in the parenthesis of Table 1, followed by the % deviation of each lattice parameter from experiments. The experimental structures in Refs. [52,53,54] were determined using X-ray diffraction at room temperature.

The SA units in Figure 1c display a packing that is influenced by hydrogen bonding between the carboxyls of each unit (mainly along *y*, but also present in *x*) and this results in a network of packed dimeric units where dispersion is greatest along *z*. DFT overestimates the lattice constants *a* and *c* by ≈ 6 and 32%, and this is improved using DFT-D; the difference between DFT-D computed lattice constants and experiment is reduced to −0.8 and −0.2% for *a* and *c*, respectively. Much like BPE, DFT-D also improves the agreement between computed and experimentally determined angle β; inclusion of dispersion changes β from 75.39${}^{\circ }$ to 93.54${}^{\circ }$, more in line with experiment.

Beyond agreement of lattice parameters, inclusion of dispersion also affects the vibrational modes of a material. We now focus on comparing DFT and DFT-D computed vibrational modes, as well as their relative contributions to the dielectric constant, ϵ, for each of the three co-crystal components, BPE, BPEth, and SA. Table 2 reports the isotropically averaged values of ϵ for DFT and DFT-D, compared to the experimentally determined values. We find that the DFT-D values are closer to experiment than DFT values, where for BPE and BPEth DFT underestimates ϵ by −22 and −14%, respectively, while for DFT-D the values are overestimated by +14 and 4%. The opposite trend is observed for SA, where DFT overestimates ϵ by −20%, and DFT-D underestimates it by 10%. Both experiment and DFT-D show that ϵ increases in the order BPE > BPEth > SA.

We now turn to an analysis of the frequencies and oscillator strength of the DFT and DFT-D computed vibrational modes that govern ϵ. Details of how to compute ϵ from first-principles using this information are given in the Materials and Methods subsection called Computational Details.

**Figure 2:**
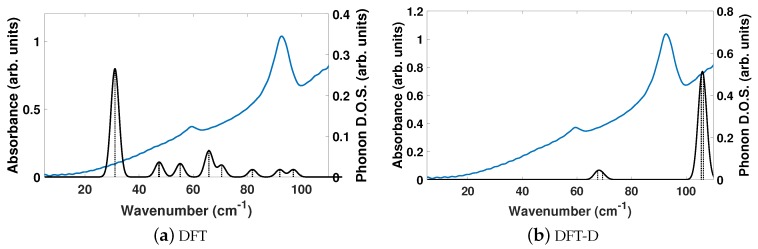
Comparisons of the computed phonon modes (black solid curves) to experimentally obtained THz spectra (solid blue curves) of BPE using (**a**) DFT and (**b**) DFT-D. Dashed black vertical lines are the normalized phonon mode intensity used to obtain the phonon density of states (DOS). All measurements are given in arbitrary units.

The experimentally determined THz spectra of BPE contains two peaks centered at 59.4 and 92.73 cm${}^{-1}$, as depicted by the blue curves in Figure 2a,b. Figure 2 is a comparison of the vibrational modes determined by an analysis of the dielectric response, as discussed in the Methodology section, between DFT (a) and DFT-D (b). We find that, in the range of 20–110 cm${}^{-1}$, DFT predicts nine total IR active modes with a contribution to ϵ of more than 0.01, and that the peak heights (scaled by contribution to ϵ in this region) are very much not in alignment. Table 3 shows that the largest contribution to ϵ in DFT is a mode at 31.1 cm${}^{-1}$, which accounts for more than 50% of the total response along *y*. This is not the case using DFT-D, which predicts four total IR active modes with a contribution larger than 0.01 to ϵ in the range of 20–110 cm${}^{-1}$. These four peaks occur as two sets of peaks at 67.6/69.5 cm${}^{-1}$ and 105.6/106.4 cm${}^{-1}$, where they are within 1–2 cm${}^{-1}$ of each other and most likely not resolvable in experiments. Figure 2b shows that DFT-D with an $s_{6}$ = 0.75 obtains two sets of modes that are shifted by +10 and +13 cm${}^{-1}$ from the experimentally determined spectra.

**Figure 3:**
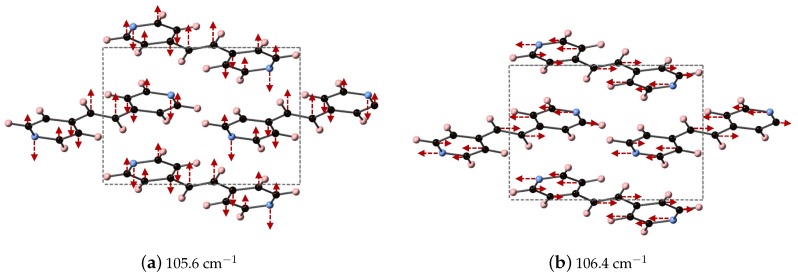
Depicted here are the DFT-D computed IR active vibrational modes of BPE that occur at (**a**) 105.6 cm^−1^ (along the *x*-direction) and (**b**) 106.4 cm^−1^ (along the *y*-direction. Color scheme is the same as before, but red arrows indicate relative atomic displacements. The displacements of H atoms are not shown for clarity.

We are able to differentiate the peaks computed by DFT-D into contributions to the *x* (*a*), *y* (*b*), and *z* (*c*) directions of ϵ, as shown in Table 3 and focus on the modes at 105.6 and 106.4 cm${}^{-1}$, which contribute ≈ 33 and 50% of the overall response in *x*- and *y*-directions, respectively. These vibrational modes are depicted in Figure 3 and can be described as asymmetric stretches where the largest displacements are localized on nitrogen and the carbon in the C${}_{2}$H${}_{2}$ unit, where these atoms displace in opposite directions.

While it is helpful to separate the components of ϵ by direction for an analysis of the vibrational modes, most experiments will report an isotropically averaged value, as discussed in the Methodology section. The DFT and DFT-D computed isotropically averaged $\epsilon_{\mu }$ (ionic response) of BPE are 0.27 and 0.29, while the directionally averaged $\epsilon_{\alpha }$ (electronic response) are 2.41 and 3.63. Inclusion of dispersion in DFT-D has increased $\epsilon_{\mu }$ by 7.4% and $\epsilon_{\alpha }$ by 50.6%. Summed together, the averaged components of ϵ for BPE using DFT and DFT-D are 2.68 and 3.92, respectively.

**Figure 4:**
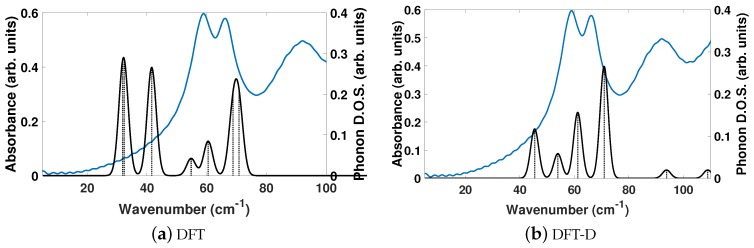
Same description as in Figure 2, but for 1,2-bis(4-pyridyl)ethane (BPEth).

The experimentally determined THz spectra of BPEth contains three peaks centered at 58.99, 66.26, and 91.92 cm${}^{-1}$, as depicted by the blue curves in Figure 4a,b. Figure 4 is a comparison of the vibrational modes determined by an analysis of the dielectric response, as discussed in the Methodology section, between DFT (a) and DFT-D (b). We find that in the range of 20–110 cm${}^{-1}$, DFT predicts seven total IR active modes with a contribution to ϵ of more than 0.01, and that the peak heights (scaled by contribution to ϵ in this region) are not in alignment, much like for BPE.

Table 4 shows that the largest contributions to ϵ in DFT are three modes below 42 cm${}^{-1}$ (at 31.8, 32.5, and 41.6 cm${}^{-1}$) and two modes at 68.8 and 70.8 cm${}^{-1}$, where all five vibrational modes have a comparable contribution to ϵ. Inclusion of dispersion in DFT-D decreases the number of IR active vibrational modes to six and changes the distribution to higher frequencies, as well as their respective contribution to ϵ. This case is depicted in Figure 4b. For example, the lowest frequency peak is at 45.4 cm${}^{-1}$, and the modes with the largest contribution to ϵ occur at 61.2 and 70.9 cm${}^{-1}$, in line with the experimentally determined spectra. Also of note in Figure 4b is the appearance of two peaks at 93.8 and 108.9 cm${}^{-1}$ in the DFT-D computed spectra, which were absent in the DFT calculation without dispersion, and coincidental with peaks in the experimentally determined THz spectra. While the DFT-D spectra of BPEth has more inconsistencies when compared to the experimental THz spectra than BPE, the frequency distribution and contribution is better than when using only DFT.

**Figure 5:**
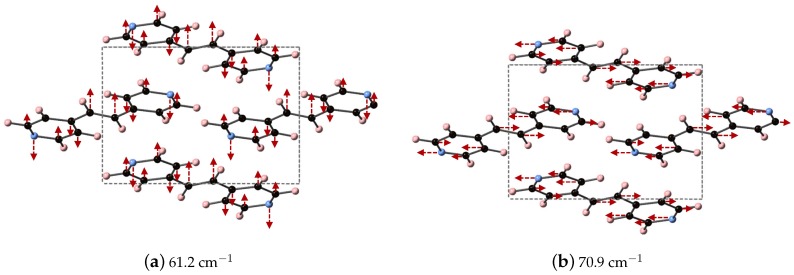
Depicted here are the DFT-D computed IR active vibrational modes of BPEth that occur at (**a**) 61.2 cm^−1^ (along the *y*-direction) and (**b**) 70.9 cm^−1^ (along the *x*-direction).

The vibrational modes of BPEth at 61.2 and 70.9 cm${}^{-1}$, which are the two largest contributors to $\epsilon_{\mu }$ in the *y*- and *x*-directions, respectively, are shown in Figure 5. The vibrational mode at 61.2 cm${}^{-1}$ can be described as an asymmetric stretch in which the largest displacements are localized on the nitrogen in the aromatic ring and the carbon in the C${}_{2}$H${}_{4}$ unit connected the two aromatic rings. Unlike the mode identified for BPE, the motion of these atoms are in the same direction, and not opposed. The vibrational mode at 70.9 cm${}^{-1}$ has displacements in the xz-direction and can be described as an asymmetric stretching mode mixed with some aromatic ring rotation. In this mode, some of the aromatic carbon displace just as far as the nitrogen and the carbon in the C${}_{2}$H${}_{4}$ unit.

The DFT and DFT-D computed isotropically averaged $\epsilon_{\mu }$ (ionic response) of BPEth are 0.32 and 0.27, while the directionally averaged $\epsilon_{\alpha }$ (electronic response) are 2.36 and 2.98. Inclusion of dispersion in DFT-D has decreased $\epsilon_{\mu }$ by 15.6% and increased $\epsilon_{\alpha }$ by 26.3%. Summed together, the averaged components of ϵ for BPEth using DFT and DFT-D are 2.68 and 3.25, respectively, comparable to the values of 2.68 and 3.92 for BPE.

Turning from the nitrogen-containing heterocycles BPE and BPEth to the carboxylic acid component of the co-crystals, we investigate the dielectric response of salicylic acid. As depicted in Figure 1c, the unit cell for SA has hydrogen bonding that takes place primarily along the *y*-axis, and this manifests as an increased contribution to $\epsilon_{\mu }$ along *y* relative to the other two axes for both DFT and DFT-D. Table 5 shows that for DFT the *y* component of $\epsilon_{\mu }$ is 1.01, while for *x* and *z* the sums are 0.79, and 0.44, respectively. Adding dispersion changes, the magnitudes of these three values, but not the trend; the trend in $\epsilon_{\mu }$ is still *y* > *x* > *z*.

**Figure 6:**
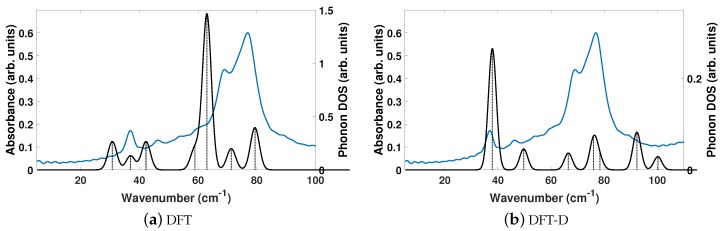
Same description as in Figure 2, but for salicylic acid (SA).

Figure 6 shows that neither DFT or DFT-D computed spectra are a good match to the experimentally determined THz spectra of SA. The seven experimentally determined THz peaks of SA are centered at 36.8, 46.1, 53.5, 60.0, 68.9, 77.0, and 89.3 cm${}^{-1}$ in the range of 20–110 cm${}^{-1}$. DFT computes too many vibrational modes at lower frequencies (30–50 cm${}^{-1}$), resulting in too many total modes, and no modes above 80 cm${}^{-1}$. DFT-D has relative peaks whose contributions to ϵ are not aligned to the experimental structure, but computed the correct number of absorption features. If we compare them to the seven DFT-D computed peaks at 37.9, 49.7, 66.5, 76.1, 78.4, 92.3, and 100.2 cm${}^{-1}$, then the differences in frequency are 1.1, 3.6, 13.0, 16.1, 9.5, 15.3, 10.9 cm${}^{-1}$ going from 20 to 110 cm${}^{-1}$.

**Figure 7:**
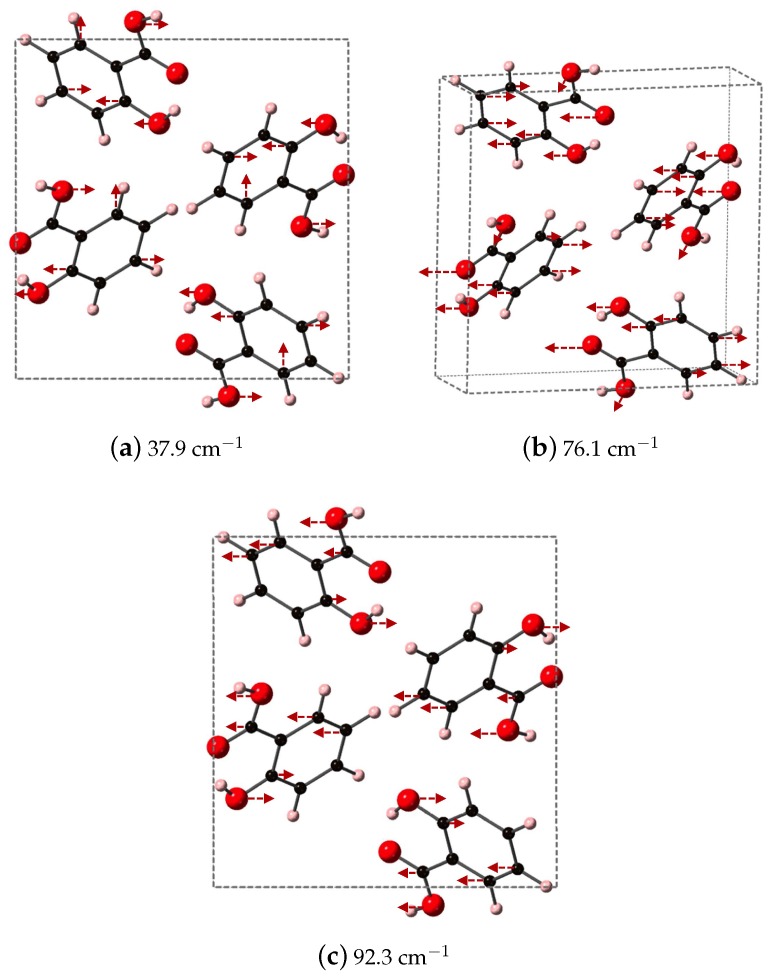
Shown here are the DFT-D computed IR active modes of SA that occur at (**a**) 37.9 cm^−1^ (along *y*); (**b**) 76.1 ^−1^ (along *xz*); and (**c**) 92.3 ^−1^ (along *y*).

We identify the three vibrational modes of SA that contribute a majority of the dielectric response, which occur at 37.9, 76.1, and 92.3 cm${}^{-1}$ and are shown in Figure 7. All three modes have significant contributions from the oxygen atoms of both the phenolic OH and COOH acid functional groups. At 37.9 cm${}^{-1}$, the OH of the COOH acid group displaces along *y*, opposite to OH on the aromatic ring, while the carbon in the aromatic ring breathes in and out. Higher in frequency, at 76.1 cm${}^{-1}$, the oxygen of the COOH carbonyl and the OH on the aromatic ring displace in the same direction along xz while carbon in the aromatic ring undergo asymmetric stretches. The highest frequency in Figure 7, 92.3 ${}^{-1}$, has the OH of the COOH acid group moving along *y*, opposed to the OH on the aromatic ring. In this vibrational mode, the carbon of CO functional group also moves, and overall the mode represents a partial rotation of SA.

For SA, we find that the difference between DFT and DFT-D computed contributions to ϵ are opposite that of BPE and BPEth; both $\epsilon_{\mu }$ and $\epsilon_{\infty }$ decrease significantly with the addition of dispersion. $\epsilon_{\mu }$ decreases from 0.75 to 0.51, and $\epsilon_{\infty }$ decreases from 2.95 to 2.28. This means that the overall averaged ϵ goes from 3.70 to 2.79, a decrease of 24.6% when including dispersion corrections. The directionally averaged value of $\epsilon_{\infty }$ (0.51) is ≈20% of the DFT-D calculated dielectric response.

In general, all three comparisons between DFT and DFT-D in Figure 2, Figure 4, and Figure 6 show that DFT will yield high-intensity, low frequency modes in the range of ≈25–40 cm${}^{-1}$ whose intensity and frequency change with the application of dispersion corrections in DFT-D. While the agreement between DFT-D and experimentally determined THz spectra is better for BPE than for BPEth and SA, DFT-D does yield results in which the three components are differentiable; BPE has IR active modes at 106 cm${}^{-1}$, BPEth has modes in the range of 60–70 cm${}^{-1}$, and SA has modes that span the range of 40–90 cm${}^{-1}$, where the modes at 38, 76, and 92 cm${}^{-1}$ all involve displacements of the oxygen in both the OH and COOH functional groups. The numerical values of dielectric response of BPE and BPEth are more similar to each other than to SA, and this may be because the only difference between them is the alkene (C${}_{2}$H${}_{2}$) vs alkane (C${}_{2}$H${}_{4}$) connection between heterocyclic rings. The contributions to $\epsilon_{\mu }$ are greatest for SA, which may be explained by the increased hydrogen bonding of SA relative to BPE and BPEth.

### 2.1. Co-Crystals

**Figure 8:**
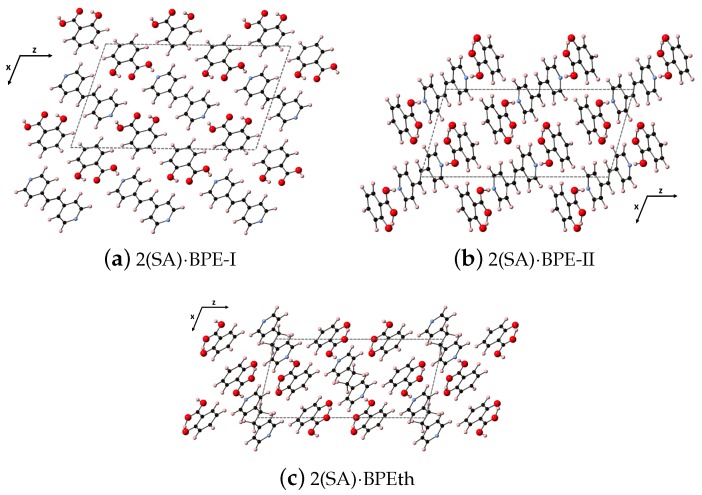
Shown here are the co-crystals (**a**) 2(SA)·BPE-I; (**b**) 2(SA)·BPE-II; and (**c**) 2(SA)·BPEth. Color scheme is as before in Figure 1.

Here, we perform the same types of analyses as in the previous section but for the larger co-crystal systems 2(SA)·BPE Forms I and II, and 2(SA)·BPEth. As will be discussed elsewhere, all three form monoclinic crystal structures (space group 14), but 2(SA)·BPE-II and 2(SA)·BPEth display packing similar to each other, while 2(SA)·BPE-I is different. The synthesis and characterization of these three co-crystals systems will be described elsewhere. While all three co-crystal structures display hydrogen bonding between the carboxyl unit of SA and the nitrogen of BPE and BPEth, 2(SA)·BPE-II and 2(SA)·BPEth also have the H of the aromatic OH of SA in close proximity to the carbonyl O of the COO unit creating a stable six member ring via resonance. This additional hybridization is absent in 2(SA)·BPE-I. The DFT-D relaxed structures are shown in Figure 8 for (a) 2(SA)·BPE-I, (b) 2(SA)·BPE-II and (c) 2(SA)·BPEth, looking down the *y*-axis at the xz plane.

We relax the co-crystal compositions using both DFT and DFT-D, and the lattice parameters of these relaxations are tabulated in Table 6. Much like the components BPE, BPEth, and SA discussed in the previous section of the Results, relaxations using DFT yield deviations from experimentally determined structures on the order of 5–25% overestimation, and the largest errors coincide with the directions where dispersion will be the greatest. This is observed for lattice constant *a* of 2(SA)·BPE-I and lattice constant *c* of 2(SA)·BPE-II and 2(SA)·BPEth. Inclusion of dispersion corrections in DFT-D decreases the error of the lattice parameters, in most cases to within the 1–2% acceptable for DFT-GGA calculations in the solid state, but DFT-D underestimates lattice constant *c* of 2(SA)·BPE-II and 2(SA)·BPEth by ≈ 8%.

**Figure 9:**
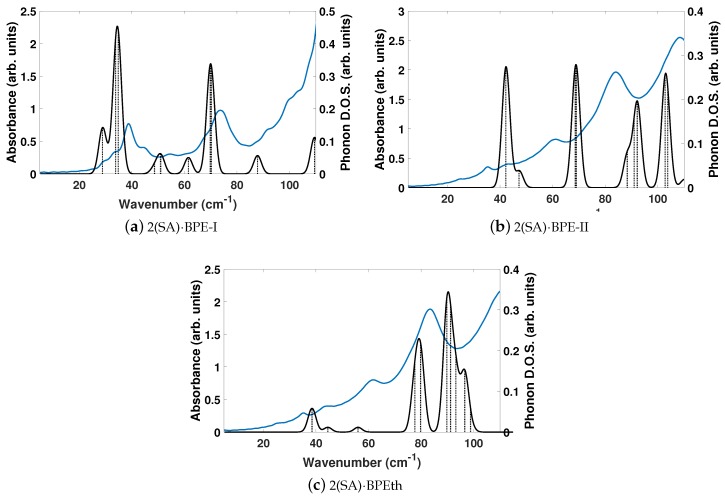
Shown here are comparisons of the computed phonon modes (black solid curves) to experimentally obtained THz spectra (solid blue curves) of (**a**) 2(SA)·BPE-I; (**b**) 2(SA)·BPE-II; and (**c**) 2(SA)·BPEth using DFT-D. Dashed black vertical lines are the normalized phonon mode intensity used to obtain the phonon density of states (DOS). All measurements are given in arbitrary units.

As the disagreement between experimentally determined and DFT-computed lattice parameters with no dispersion correction is significantly large, and we showed earlier that DFT-D computed vibrational modes of the components were needed for a better description of the THz spectral features, we compute the vibrational modes of the co-crystals using DFT-D. Figure 9 depicts the DFT-D computed vibrational modes of the co-crystals, compared to experimentally determined THz spectra. The comparison of vibrational modes in Figure 9a shows that DFT-D predicts low frequency modes for 2(SA)·BPE-I (below 40 cm${}^{-1}$) close to experiment. Experiment shows ten THz peaks of 2(SA)·BPE-I centered at 29.5, 33.5, 38.8, 44.9, 54.6, 68.1, 73.7, 92.7, 99.6, and 102.4 cm${}^{-1}$, compared to DFT-D which predicts IR active modes at 28.9, 33.9, 34.9, 48.5, 50.9/51.1, 61.5, 69.8/70.3, 87.8, 109.5 cm${}^{-1}$. The smallest difference in frequencies between THz spectroscopy and DFT-D is on the order of 1–2 cm${}^{-1}$ and the largest difference in frequencies is on the order of 10 cm${}^{-1}$. The minor deviations in vibrational mode frequencies, from the experimentally determined values, may be related to the close agreement between experimentally determined and DFT-D computed lattice parameters, which are on the order of ± 1.5%. The experimentally determined THz spectra of 2(SA)·BPE-II (Figure 9b) and 2(SA)·BPEth (Figure 9c) are similar with frequencies centered at 35.2, 43.6, 61.6, 83.4, and 110.7 cm${}^{-1}$ for 2(SA)·BPEth and at 24.9, 35.4, 42.8, 60.8, 84.0, and 108.7 cm${}^{-1}$ for 2(SA)·BPE-II. The experimentally determined frequencies are listed in Table 7.

**Figure 10:**
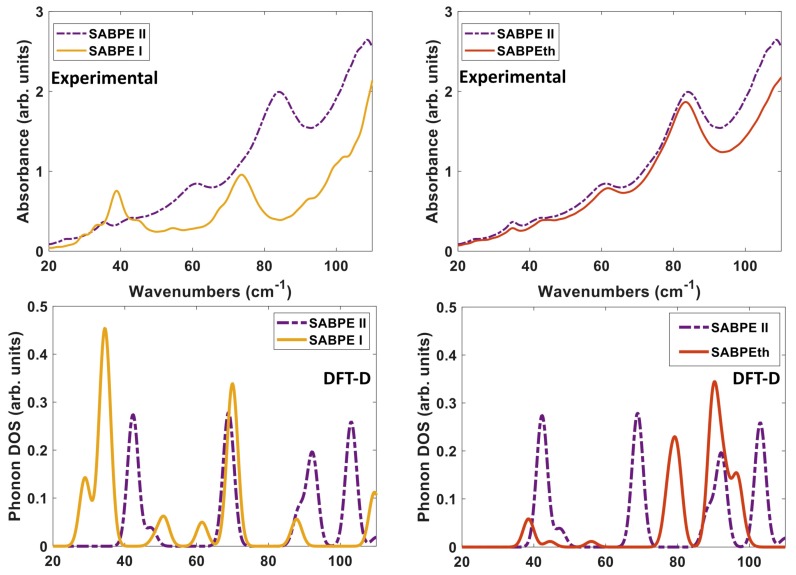
Comparisons of the Experimental THz spectra (**top**) and DFT-D computed phonon modes (**bottom**) of (**left**) 2(SA)·BPE-I (solid yellow line) and 2(SA)·BPE-II (dash-dotted purple line), and (**right**) 2(SA)·BPE-II (dash-dotted purple line) and 2(SA)·BPEth (solid orange line).

We compare both the experimentally determined and DFT-D computed spectra of the three co-crystals to each other in Figure 10. When comparing the experimental spectra (top of Figure 10), it seems that orientation and packing effects dominate the THz moreso than the difference in ligand identity as BPE and BPEth. The comparison of 2(SA)·BPE-I and 2(SA)·BPE-II in Figure 10 (left) highlights the difference between co-crystals with the same components, but different crystal packing and, thus, different lattice parameters. The experimental spectra show coincidental peaks only at ≈35 and 43 cm${}^{-1}$, while the DFT-D do not coincide at low frequency (30–60 cm${}^{-1}$), coincide in the range of 70–90 cm${}^{-1}$, and begin to differ again above 90 cm${}^{-1}$.

For 2(SA)·BPE-II and 2(SA)·BPEth, the DFT-D calculated IR active modes are consistently shifted higher, at least 5–8 cm${}^{-1}$ than experiments, if they match up at all. This mismatch between the THz and DFT-D for 2(SA)·BPEth and 2(SA)·BPE-II co-crystals may be caused by the ≈ 8% underestimation of lattice constant *c*. Figure 10 (right) shows that the experimental THz spectra of 2(SA)·BPE-II and 2(SA)·BPEth coincide at all frequencies above 30 cm${}^{-1}$ and that for the DFT-D computed spectra they (roughly) coincide at low frequencies (30–60 cm${}^{-1}$), do not coincide in the range of 70–90 cm${}^{-1}$, and begin to coincide again above 90 cm${}^{-1}$.

**Figure 11:**
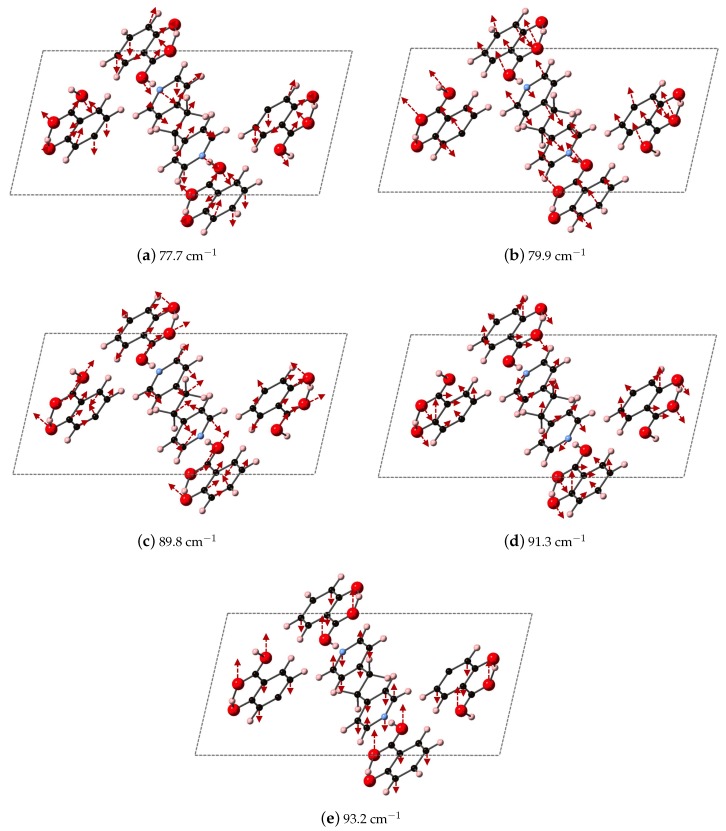
Shown here are the DFT-D computed IR active modes of 2(SA)·BPEth that occur at (**a**) 77.7 cm^−1^ (along *xz*); (**b**) 79.9 cm^−1^ (along *y*); (**c**) 89.8 cm^−1^ (along *xz*); (**d**) 91.3 cm^−1^ (along *y*); and (**e**) 93.2 cm^−1^ (along *xz*).

**Figure 12:**
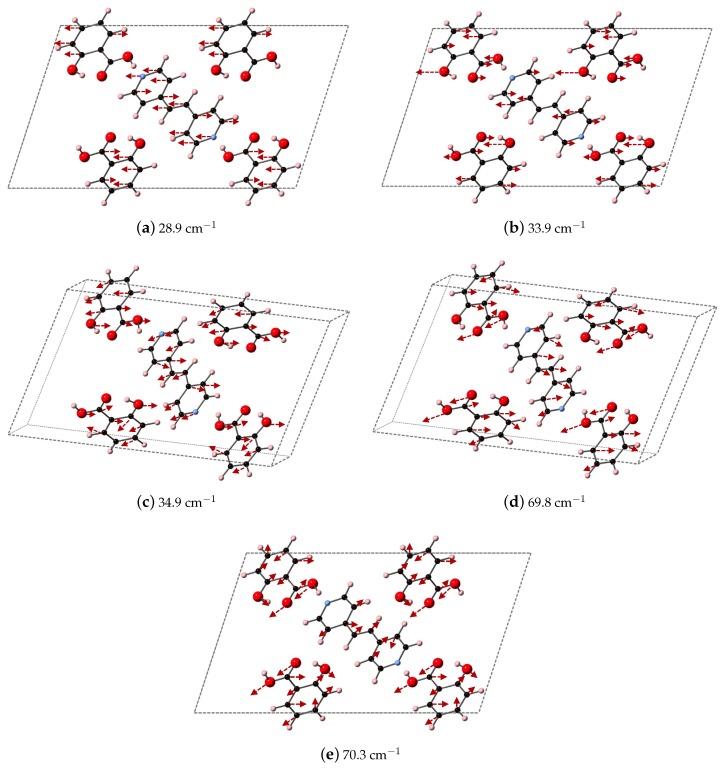
Plotted here are the DFT-D computed IR active modes of 2(SA)·BPE-I that occur at (**a**) 28.9 cm^−1^ (along *xz*); (**b**) 33.9 cm^−1^ (along *xz*); (**c**) 34.9 cm^−1^ (along *y*); (**d**) 69.8 cm^−1^ (along *y*); and (**e**) 70.3 cm^−1^ (along *xz*).

**Figure 13:**
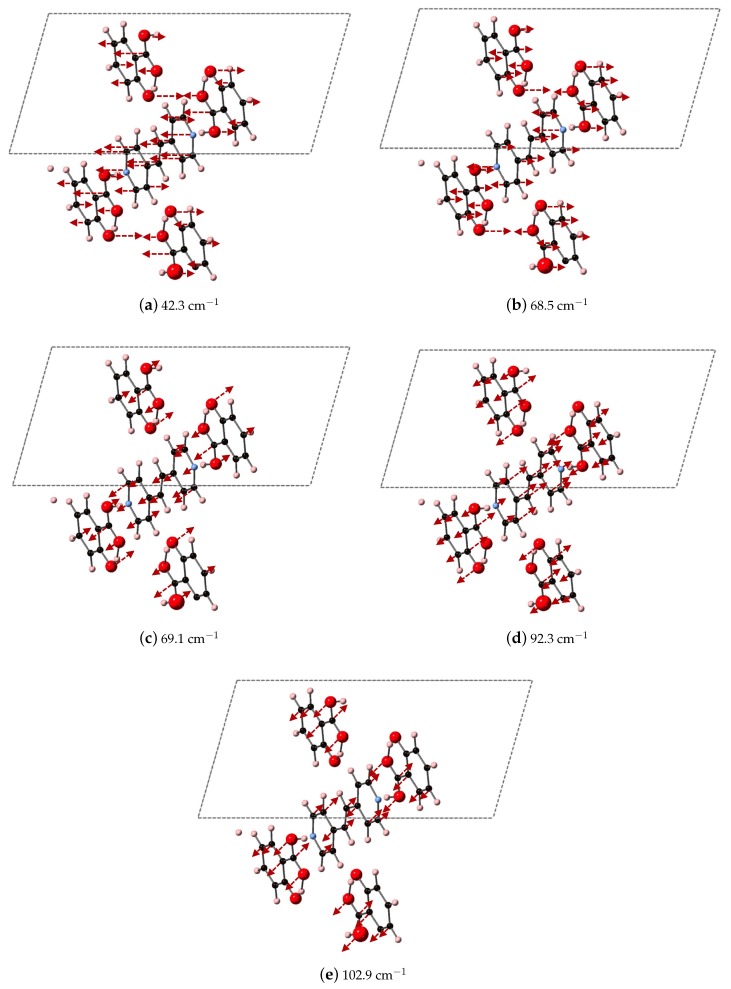
Depicted here are the DFT-D computed IR active modes of 2(SA)·BPE-II that occur at (**a**) 42.3 cm^−1^ (along *xz*); (**b**) 68.5 cm^−1^ (along *z*); (**c**) 69.1 cm^−1^ (along *y*); (**d**) 92.3 cm^−1^ (along *xz*); and (**e**) 102.9 cm^−1^ (along *xz*).

We compare the three ranges of frequencies computed via DFT-D to determine if our methods could yield spectral resolution that goes beyond the resolution capabilities of experimental THz data collection at room temperature, and our analysis points towards characteristic frequency ranges for these co-crystal systems that may indicate where differences in packing vs. differences in bipyridyl coformer (as ligand identity) will dictate vibrational modes. We find that packing arrangement may dominate between 30–60 cm${}^{-1}$, ligand substitutions may dominate in the range of 70–90 cm${}^{-1}$, and then packing may dominate again above 90 cm${}^{-1}$. Example vibrational modes computed using DFT-D in these three regions are shown in Figure 11, Figure 12 and Figure 13 for 2(SA)·BPEth, 2(SA)·BPE-I, and 2(SA)·BPE-II, respectively. The differences between the the experimentally determined and DFT-D computed vibrational modes motivate the need for further investigations that could include low temperature THz spectra collection and methodology that goes beyond the Grimme D2 formalism used here, which will be discussed in the next section.

The DFT-D calculated contributions to $\epsilon_{\mu }$ for 2(SA)·BPEth are tabulated in Table 8 and Table 9 for 2(SA)·BPE polymorphs I and II. The directionally averaged values of $\epsilon_{\mu }$ and $\epsilon_{\infty }$ for 2(SA)·BPEth are 1.14 and 2.97, 1.48 and 3.05 for 2(SA)·BPE-I, and 0.81 and 3.33 for 2(SA)·BPE-II, and when added together yield ϵ that are 4.11, 4.53, and 4.14, respectively. $\epsilon_{\mu }$ for the co-crystals is 20–30% of the total dielectric response, an increase when compared to the behaviors of their components, such as SA (18%) and BPEth (8%). The isotropically averaged values of ϵ for the three co-crystals are tabulated in Table 10, compared to the experimentally determined values. We find that all three DFT-D computed ϵ are underestimated compared to experiments, and that, unlike the ϵ of the components, the trends in magnitude of ϵ do not agree between experiments and DFT-D. Moreover, the DFT-D calculated values are closer to each other than those measured experimentally.

The overall dielectric responses of the co-crystals and their components also demonstrate that intermolecular interactions, highly dependent on packing orientation, result in a dielectric response that is not merely an average of the components. For example, the directionally averaged values of $\epsilon_{\mu }$ for SA and BPE are 0.51 and 0.29, respectively, and an average of those two numbers is 0.40. The values for 2(SA)·BPE-I and 2(SA)·BPE-II are 1.48 and 0.81, which is more than even when the $\epsilon_{\mu }$ are added together (0.79). This implies a tunable, nonlinear dielectric response can be achieved, and potentially optimized, in co-crystal formulations.

Analysis of the vibrational modes of the co-crystals show that all modes in the range of 20–110 cm${}^{-1}$ have significant contributions from both the nitrogen containing heterocycles BPE and BPEth, and SA. A majority of the largest displacements in each mode above 40 cm${}^{-1}$ is localized on both of the O atoms of the COOH acid, and the phenolic OH of SA, and in many cases the nitrogen of the heterocyclic ring as well. Below 40 cm${}^{-1}$, such as the modes of 2(SA)·BPE-I in Figure 12, O motion contributes less than the motions present in the heterocyclic ring. Vibrational modes with frequencies higher than 90 cm${}^{-1}$, such as the modes of 2(SA)·BPE-II in Figure 13 also tend to have larger carbon displacements than modes at lower frequencies.

### 2.2. Improving Agreement with Experiment

In the two previous sections of the Results and Discussion, we showed that using DFT-D to compute the lattice parameters and vibrational spectra of the 2(SA)·BPE and 2(SA)·BPEth co-crystals, as well as their components, results in a marked improvement over the same parameters computed using DFT. There still remains some inconsistencies between DFT-D computed spectra and experimentally determined lattice parameters and THz spectra, such as 8% lattice constant underestimation for 2(SA)·BPE-II and 2(SA)·BPEth and the upshift in frequency for the IR active modes of BPE.

One method to improve agreement between experimental THz spectra and DFT-D modeling is to adjust the global dispersion scaling factor, $s_{6}$, from the default value of 0.75. Systematically changing $s_{6}$ results in changes in lattice parameters and frequencies of IR-active modes, where better agreement can be reached between modeling efforts and THz spectra [4,18,19,55]. Here, we turn to another open source planewave DFT code in which varying $s_{6}$ is allowed in the input file. We use Quantum Espresso [35] and the GBRV (Garrity-Bennett-Rabe-Vanderbilt) [29] set of ultrasoft pseudopotentials [27] to map out how changing the global dispersion correction will affect lattice parameters for BPE, BPEth, SA, and 2(SA)·BPEth. We keep the *k*-grid sampling and energy convergence criteria the same as for the calculations employing ABINIT [34] and the ONCV-type (optimized norm-conserving Vanderbilt) of pseudopotential [28].

**Figure 14:**
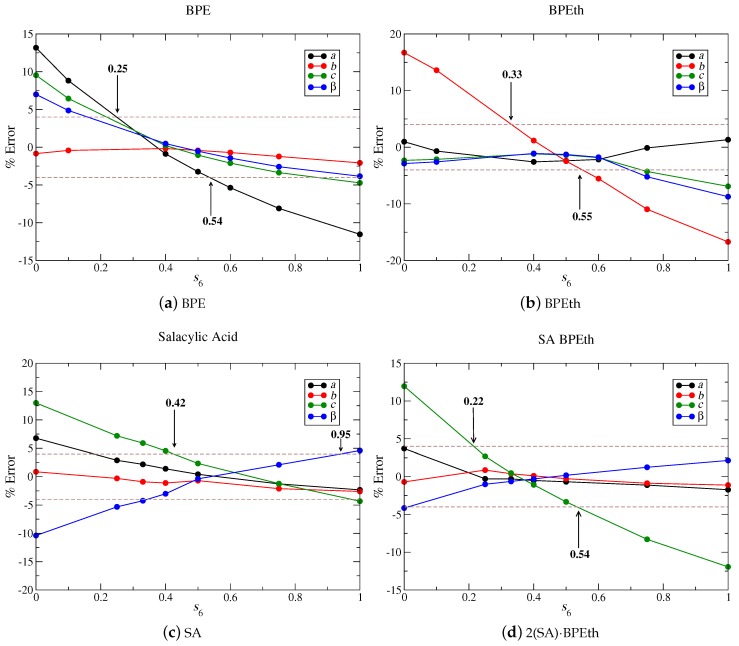
For co-crystal components (**a**) BPE; (**b**) BPEth; and (**c**) SA, we vary *s*_6_ from 0 to 1 to determine how DFT-D computed lattice parameters *a*, *b*, *c*, and *β* will be affected when compared to experimentally determined lattice parameters. The same tests for co-crystal 2(SA)·BPEth yield a similar range of *s*_6_ in which lattice constant underestimation may be minimized. Brown dashed lines denote ±4% error with respect to experimentally determined lattice parameters.

Figure 14 shows that $s_{6}$ values of 0.4, 0.5, 0.6, and 0.35 for BPE (a), BPEth (b), SA (c), and 2(SA)·BPEth (d) independently minimize the percent error when lattice parameters are compared to experiment. If we increase the maximum % error to ± 4%, then we obtain ranges of $s_{6}$ values of 0.25–0.54 for BPE, 0.33–0.55 for BPEth, 0.42–0.95 for SA, and 0.22–0.54 for 2(SA)·BPEth. In all cases, but SA, an $s_{6}$ = 0.75 is not present in any of these ranges. Using these four test cases as an example, we obtain a range of 0.42 < $s_{6}$ < 0.54, which is in line with the values used in previous studies of organic co-crystals [4,18,19,55].

## 3. Materials and Methods

### 3.1. Pellet Fabrication

Pellets were prepared by the methods described before [56] for the single components as well as the co-crystals, of which the details of syntheses and complete structural characterization will be detailed elsewhere. Briefly, a commercial PTFE (Polytetrafluoroethylene) powder, with particle diameters ranging from 9–13 μm, was purchased from Micro Powders (FLUO 625 CTX2, Micro Powders, INC., New York, NY, USA). The powder was dried at 60 ${}^{\circ }$C and stored in a desiccator before use. For each analyte, a mixture was prepared by co-grinding 40 mg of analyte with 1700 mg of dried PTFE for five minutes in an agate mortar and pestle. Three sample pellets were prepared from each mixture by placing 400–450 mg of the mixture into a 13 mm diameter stainless steel die and using a Specac hydraulic press (model number 15011, Kent, UK) to apply a 5-ton load (0.34 GPa) for 5 min. Freshly formed sample pellets were removed from the die and placed in a desiccator until spectra were collected.

### 3.2. Dielectric Measurements

Dielectric constants were extracted as described previously [57]. Briefly, values of refractive index (η) are extracted from phase information embedded within the time-domain spectra.

The refractive index of the pellet is related to the difference in phases for the sample pellet ($\phi_{s}$) and reference air ($\phi_{r}$), according to Equation (1), where *c* is the speed of light, *b* is the sample thickness, or path length, and ω is the frequency of the electromagnetic radiation in Hz. The dielectric constant for the sample can be obtained according to Equation (2), where ϵ(ν) represents the dielectric constant as a function of frequency in wavenumber and *k*(ν) is the frequency dependent extinction coefficient for the pellet material. Generally, *k*(ν) is negligible [58] and the dielectric constant can be taken as the square of the refractive index:

(1) $$\begin{array}{c} \eta \left(\omega \right)=1-\frac{c}{2\pi \omega b}\left(\phi_{s}\left(\omega \right)-\phi_{r}\left(\omega \right)\right), \end{array}$$

(2) $$\begin{array}{c} \epsilon \left(\nu \right)=\eta {\left(\nu \right)}^{2}+k{\left(\nu \right)}^{2}. \end{array}$$

As the pellets are mostly composed of PTFE, the analyte dielectric must be extracted using the Landau, Lifshitz and Looyenga (LLL) model [59]. This model is defined in Equation (3) using the Looyenga power law, specifically for our three-component system. In Equation (3), the dielectric constant of the pellet is composed of a volume fraction (*v* of each component; PTFE, sample, and air). In this work, the volume of air is obtained by subtracting the total volume of the pellet from the sum of volumes of the crystals and PTFE. The volumes of both the PTFE and crystals were determined from the mass of each and their known densities. For the crystalline samples examined here, the crystal structure density was used and, for PTFE, the value of 2.26 g/cm${}^{3}$ was used for its density. The density of PTFE was measured using the volume and mass of a pure pellet:

(3) $$\begin{array}{c} \epsilon^{1/3}=\epsilon_{\mathrm{air}}^{\prime 1/3}v_{\mathrm{air}}+\epsilon_{\mathrm{PTFE}}^{\prime 1/3}v_{\mathrm{PTFE}}+\epsilon_{\mathrm{analyte}}^{\prime 1/3}v_{\mathrm{analyte}}. \end{array}$$

### 3.3. Terahertz Spectroscopy

THz transmission spectra were collected and analyzed by the methods described before [56] using with a Teraview TPS 1000D time-domain terahertz spectrometer (TeraView Limited, Cambridge, UK). For each sample, three pellets were tested by rotating the samples into the sample holder three times and taking three triplicate measurements. This resulted in a total of 27 time-domain spectra for each analyte. Between each consecutive measurement, an air background was collected. Each spectrum was collected as 1800 co-added scans attained over one minute. Purging the sample compartment with dried air avoided the presence of confounding water vapor lines.

Time-domain spectra were truncated just prior to the etalon feature and zero-filled to 8192 (2${}^{13}$) points. The truncated data were treated with a boxcar apodization function followed by the Fourier transformation to yield the corresponding frequency-domain electric field spectrum. Absorbance spectra were then calculated as twice the negative base ten logarithm of the ratio of the sample to air electric field spectra [60]. Twice the negative base ten logarithm is required to square the electric field values in order to realize intensities. The resolution of the resulting spectra was 1.2 cm${}^{-1}$ over a spectral range of 10–110 cm${}^{-1}$.

### 3.4. Computational Details

Density functional theory (DFT) calculations with periodic repeat boundary conditions were carried out using the ABINIT open source software package [34], unless otherwise noted. The generalized gradient approximation (GGA) of Perdew, Burke and Ernzerhof (PBE) [32] was used as the exchange-correlation functional for all calculations, and DFT structural optimizations that included dispersion corrections used the Grimme-D2 implementation [43]. All structural optimizations employed a variable cell relaxation where all lattice constants, lattice angles, and Wyckoff positions were optimized concurrently starting from the experimentally determined monoclinic crystals, and monoclinic symmetry was maintained throughout all structural relaxations. We differentiate between the two types of calculations as DFT (with no dispersion correction) and DFT-D (which includes dispersion correction). For all DFT-D calculations, the functional dependent scaling factor, $s_{6}$, was 0.75, unless otherwise noted.

All atoms were represented as optimized norm-conserving Vanderbilt (ONCV) pseudopotentials [28], that were chosen because of their accuracy and computational efficiency [30]. All calculations used a plane wave cutoff of 40 Ry, and all atoms were allowed to fully relax during structural optimizations. The convergence criteria for structural optimizations was a maximum residual force of 5 meV/Angstrom per atom. The input structures for the dispersion corrected DFT calculations will be detailed elsewhere. A 4 ×4 × 4 *k*-point mesh [61] was used for all structural optimizations because the difference in total energy between a 4 × 4 × 4 and 6 × 6 × 6 *k*-point mesh was below 3 meV per co-former unit.

Here, we follow the methodology outlined for first-principles calculations of static dielectric properties [62,63] as described in previous work on solid state bulk materials [64,65,66]. Briefly, once a structure is fully relaxed using either DFT or DFT-D, response function calculations [67,68] are carried out to generate $D_{\alpha \beta }\left(i,j\right)$, the mass weighted dynamical matrix:

(4) $$\begin{array}{c} D_{\alpha \beta }\left(i,j\right)=\frac{\partial^{2}E}{\sqrt{m_{i}m_{j}}\partial \tau_{i\alpha }\partial \tau_{j\beta }}. \end{array}$$

In Equation (4), *E* is the DFT (or DFT-D) computed total energy, $m_{i}$ ($m_{j}$) is the mass of atom *i* (*j*), and $\tau_{i\alpha }$ is the displacement of atom *i* (*j*) in direction α (β). This creates a square matrix of dimension 3*N*×3*N*, where *N* is the total number of atoms, and each eigenvalue of matrix *D* is $\omega_{\mu }^{2}$, the frequency squared of a normalized eigenvector $a_{\mu }$. The means that the square root of the eigenvalues are the frequencies that we report in cm${}^{-1}$, and its eigenvector is a vibrational mode. For each atom, the Born effective charge tensor, $Z_{i\alpha \beta }^{*}$, is also calculated and used to compute the effective charge for each mode μ. The mode effective charge, $Z_{\mu \alpha }^{*}$, is defined as:

(5) $$\begin{array}{c} Z_{\mu \alpha }^{*}=\sum_{i\beta }\frac{Z_{i\alpha \beta }^{*}{\left(a_{\mu }\right)}_{i\beta }}{\sqrt{m_{i}}}. \end{array}$$

Contributions to the static dielectric response come only from vibrational modes that are IR active, and thus have a non-zero $Z_{\mu \alpha }^{*}$. It is these modes that are used to calculate $\epsilon_{\mu }$, the ionic contribution to the dielectric constant from mode μ using the following equation:

(6) $$\begin{array}{c} \epsilon_{\mu \alpha \beta }=\frac{Z_{\mu \alpha }^{*}Z_{\mu \beta }^{*}}{4\pi^{2}\epsilon_{0}V\omega_{\mu }^{2}}, \end{array}$$

where $\epsilon_{0}$ is the permittivity of free space and *V* is the volume of the bulk solid. The total (isotropically averaged) ionic contribution to the dielectric constant is therefore given by:

(7) $$\begin{array}{c} \epsilon_{\mu }=\frac{1}{3}\sum_{\alpha }\epsilon_{\mu \alpha \alpha }. \end{array}$$

In addition to computing $\omega_{\mu }$, $a_{\mu }$, and $Z_{i\alpha \beta }^{*}$, the response function capabilities of ABINIT can also produce $\epsilon_{\infty }$, which is the directionally averaged electronic contribution to the dielectric tensor. This is sometimes referred to as the high-field limit response, where the application of a high frequency alternating electric field prevents ionic motion and any response is purely electronic in nature. This separation in behavior, ionic vs. electric, means that the total static dielectric response can therefore be written as:

(8) $$\begin{array}{c} \epsilon =\epsilon_{\infty }+\sum_{\mu }\epsilon_{\mu }. \end{array}$$

Here, we focus on characterizing the IR-active modes in the region of 20–110 cm${}^{-1}$, in line with the collected THz data, with meaningful contributions to the dielectric response. For all comparative IR active mode plots, the DFT-computed spectral features are generated using a Gaussian distribution with a full width at half max of 1.5 cm${}^{-1}$. The relative DFT-computed peak intensities, plotted as a phonon density of states (DOS), are determined by their specific contributions to ϵ in the range of 20–110 cm${}^{-1}$, which is specified for the spectroscopic analysis presented here.

## 4. Conclusions

The comparison of DFT to DFT-D for the organic compounds BPE, BPEth, and SA shows that the inclusion of dispersion using the semiempirical Grimme-D2 dispersion correction will yield lattice parameters and vibrational modes whose values are closer to those measured in experiment. We find that the agreement between DFT-D computed vibrational modes and THz spectra was best for BPE and worst for SA. As pointed out in the final subsection of the Results and Discussion, this may be caused by using an $s_{6}$ global scaling factor that was too large, 0.75 vs. ≈0.50, or could also be because of the increased amount of hydrogen bonding in the SA crystal structure. With regards to the co-crystals 2(SA)·BPEth and 2(SA)·BPE forms I and II, DFT-D did pick out differentiable trends between the systems, but the lattice constant disagreement (for 2(SA)·BPEth and 2(SA)·BPE-II) between theory and experiment was still too large to be able to assign specific vibrational features to the THz spectra. Moreover, the experimental spectra showed that atomic packing and overall crystal lattice structure dominated the THz region moreso than changes in ligand identity as BPE vs. BPEth, while DFT-D showed that both atomic packing and ligand identity led to differentiable regions where each tended to dominate. One way to obtain more information about the DFT-D computed vibrational modes, and to match more closely to experiments, would be to use what is presented here as input for molecular dynamics calculations that take into account effects such as temperature or by quasi-harmonic DFT approximations [69] that map out the effects of temperature and volume changes on solid-state properties related to low frequency vibrations [70,71,72].

Our study determines the optimal ranges of $s_{6}$ for the set of materials under investigation and we are able to discern differences in $s_{6}$ that may be correlated to crystal structure/property relations and be used as a guide for further investigations. Organic heterocycles like BPE and BPEth with π–π stacking interactions can be optimized with $s_{6}$ ≈ 0.3–0.5, and organics that demonstrate significant H-bonding, like SA, can be optimized with $s_{6}$ ≈ 0.4–0.9. Comparing the $s_{6}$ of the combination, these two different types of bonding schemes (to create co-crystals) also yield insights; the lattice parameters of 2(SA)·BPEth co-crystal are closest to experiments when $s_{6}$ approaches the values used for organic materials with π–π stacking and not those with significant hydrogen-bonding. This suggests that, in complex materials, containing multiple functional groups or packing behavior, at least two or three different $s_{6}$ should be used for preliminary structural optimizations. Determining which range of $s_{6}$ may be best will allow for a better understanding of the relative forces that stabilize a complex structure.

Our analysis of the dielectric response of BPE, BPEth, and SA shows that functional group identity, degree of hydrogen bonding, and crystal packing/arrangement will influence the dielectric constant, ϵ. This work could be extended to analyze and compare the effects of fluoride, cyano, aldehyde, and amide substituents on aromatic heterocycles similar to what is presented here, or on different types of heterocycles (pyrazine, imidazole, furan, thiophene) and 3D bonding architectures (fullerenes, substituted adamantanes) on dielectric behavior, to be used as a design parameter for co-crystal APIs.

Here, we establish a baseline of DFT computed dielectric response and THz spectroscopy for the organic co-crystals 2(SA)·BPEth and 2(SA)·BPE forms I and II, as well as their constituent components, using dispersion corrected planewave DFT methods that employ the GBRV set of ultrasoft pseudopotentials. Further development of the work presented here could include using our Grimme-D2 results as a starting point for calculations that take into account molecular environments, such as the parameter free approach developed by Tkatchenko and Scheffler [73] or the Grimme-D3 methodology, which employs a three-body dispersion term [74] and fractional coordination numbers. Recently developed dispersion corrected hybrid functionals [75,76] may also improve the agreement between DFT modeling and THz measurements beyond what is presented here using a combination of DFT-GGA and Grimme-D2 dispersion correction.

## Figures and Tables

**Table 1:** Lattice parameters of co-crystal components BPE, BPEth, and SA, using DFT (top) and DFT-D (bottom). All lattice constants are reported in units of Å, and both the experimental value and % deviation from experimentally determined values are given in parentheses.

	$a_{\mathrm{DFT}}$	$b_{\mathrm{DFT}}$	$c_{\mathrm{DFT}}$	$\beta_{\mathrm{DFT}}$
	(Å)	(Å)	(Å)	
BPE	11.00 (7.82, +40.66)	10.38 (10.56, −1.70)	6.89 (5.77, +19.41)	116.89 (92.68, +26.12)
BPEth	5.69 (5.56, +2.33)	9.64 (8.16, +18.14)	11.33 (11.35, −0.18)	99.26 (100.73, −1.45)
SA	5.20 (4.89, +6.38)	11.02 (11.20, −1.64)	14.83 (11.24, +31.94)	75.39 (92.49, −18.49)
BPE	7.30 (7.82, −6.66)	10.51 (10.56, −0.50)	5.56 (5.77, −2.87)	90.39 (92.68, −2.47)
BPEth	5.31 (5.56, −4.53)	7.80 (8.16, −4.38)	11.11 (11.35, −2.16)	98.73 (100.73, −1.99)
SA	4.85 (4.89, −0.78)	11.02 (11.20, −1.64)	11.27 (11.24, +0.23)	93.54 (92.49, +1.14)

**Table 2:** Comparison of experimentally determined dielectric constant, ϵ, to the isotropically averaged values computed using DFT and DFT-D for the co-crystal components BPE, BPEth, and SA. Percent deviation from experiment is shown in parenthesis for DFT and DFT-D.

	Experiment	DFT	DFT-D
BPE	3.43 ± 0.08	2.68 (−21.87)	3.92 (+14.29)
BPEth	3.13 ± 0.15	2.68 (−14.38)	3.25 (+3.83)
SA	3.09 ± 0.04	3.70 (+19.74)	2.79 (−9.71)

**Table 3:** Mode-by-mode analysis of the directional components of the IR active response of BPE for DFT (top) and DFT-D (bottom) for the THz frequency range of 20–110 cm${}^{-1}$. ω are given in units of cm${}^{-1}$ and all ϵ are unitless. Asterisks (*) are next to the frequencies with high contributions to $\epsilon_{\mu }$, and are shown in Figure 3. The two final rows in each of the DFT and DFT-D calculations are the total ionic portion of the dielectric response per direction *x*, *y*, *z* per mode μ (denoted as $\epsilon_{\mu }$), and the directionally decomposed electronic contribution of the dielectric response $\epsilon_{\infty }$.

Method	ω	$\epsilon_{x}$	$\epsilon_{y}$	$\epsilon_{z}$
DFT	31.1	-	0.29	-
	47.2	-	0.01	-
	47.5	-	0.03	-
	55.2	0.02	-	0.03
	65.8	-	0.07	-
	70.5	0.01	-	0.03
	81.9	0.02	-	0.00
	92.0	-	0.06	-
	97.0	0.02	-	0.00
$\epsilon_{\mu }$		0.12	0.55	0.15
$\epsilon_{\infty }$		1.97	2.86	2.40
DFT-D	67.6	-	0.02	-
	69.5	0.01	-	0.00
	105.6 *	0.15	-	0.00
	106.4 *	-	0.15	-
$\epsilon_{\mu }$		0.44	0.30	0.14
$\epsilon_{\infty }$		2.63	4.63	3.62

**Table 4:** Same description as Table 3, but for BPEth. Asterisks (*) are next to the vibrational modes shown in Figure 5.

Method	ω	*x*	*y*	*z*
DFT	31.8	-	0.11	-
	32.5	-	0.10	-
	41.6	0.10	-	0.16
	54.8	-	0.03	-
	60.5	-	0.06	-
	68.8	0.10	-	0.01
	70.8	-	0.11	-
$\epsilon_{\mu }$		0.26	0.48	0.22
$\epsilon_{\infty }$		2.20	2.18	2.69
DFT-D	45.4	-	0.06	-
	53.9	0.03	-	0.00
	61.2 *	-	0.08	-
	70.9 *	0.13	-	0.04
	93.8	-	0.01	-
	108.9	0.00	-	0.01
$\epsilon_{\mu }$		0.24	0.43	0.13
$\epsilon_{\infty }$		2.97	2.55	3.43

**Table 5:** Same description as Table 3, but for SA. Asterisks (*) denote the modes depicted in Figure 7.

Method	ω	*x*	*y*	*z*
DFT	30.8	-	0.04	-
	37.0	0.02	-	0.00
	42.2	-	0.02	-
	42.3	0.02	-	0.00
	58.9	0.03	-	0.00
	63.0	-	0.22	-
	71.3	0.03	-	0.00
	79.2	-	0.04	-
	79.5	0.09	-	0.00
$\epsilon_{\mu }$		0.79	1.01	0.44
$\epsilon_{\infty }$		3.00	3.11	2.73
DFT-D	37.9 *	-	0.29	-
	49.7	0.05	-	0.00
	66.5	-	0.04	-
	76.1 *	0.07	-	0.03
	78.4	-	0.02	-
	92.3 *	-	0.09	-
	100.2	0.03	-	0.01
$\epsilon_{\mu }$		0.55	0.69	0.28
$\epsilon_{\infty }$		2.19	2.32	2.34

**Table 6:** Lattice parameters of co-crystals using DFT (top) and DFT-D (bottom). All lattice constants are reported in units of Åand % deviation from experimentally determined values are given in parentheses after the experimentally determined values.

Co-Crystal	$a_{\mathrm{DFT}}$	$b_{\mathrm{DFT}}$	$c_{\mathrm{DFT}}$	$\beta_{\mathrm{DFT}}$
	(Å)	(Å)	(Å)	
2(SA)·BPE-I	13.48 (11.93, +12.99)	4.89 (4.87, +0.45)	20.71 (20.25, −2.27)	99.81 (106.92, −6.65)
2(SA)·BPE-II	9.12 (8.76, +4.11)	6.48 (6.81, −4.85)	24.65 (19.66, +25.38)	110.86 (105.34, +5.24)
2(SA)·BPEth	9.25 (8.63, +7.18)	6.49 (6.86, −5.39)	22.35 (19.55, +14.32)	91.94 (101.36, −9.29)
2(SA)·BPE-I	11.76 (11.93, −1.41)	4.89 (4.87, +0.45)	19.89 (20.25, −1.08)	108.07 (106.92, +1.08)
2(SA)·BPE-II	8.73 (8.76, −0.34)	6.87 (6.81, +0.91)	18.06 (19.66, −8.15)	105.91 (105.34, +0.54)
2(SA)·BPEth	8.61 (8.63, −0.26)	6.88 (6.86, +0.34)	17.98 (19.55, −8.02)	102.92 (101.36, +1.54)

**Table 7:** Experimentally determined THz frequencies for the co-crystals 2(SA)·BPE form I and II, and 2(SA)·BPEth, given in units of cm${}^{-1}$. Values are partitioned to highlight similarities and differences.

2(SA)·BPE-I	29.5	33.5	38.8	44.9	54.6		68.1	73.7		92.7	99.6	102.4
2(SA)·BPE-II	24.9	35.4		42.8		60.8			84.0			108.7
2(SA)·BPEth		35.2		43.6		61.6			83.4			110.7

**Table 8:** Mode-by-mode analysis of the directional components of the IR active response of 2(SA)·BPEth co-crystal using DFT-D for the THz frequency range of 20–110 cm${}^{-1}$. ω are given in units of cm${}^{-1}$ and all ϵ are unitless. Asterisks are next to the frequencies that have high contributions to $\epsilon_{\mu }$ and are plotted in Figure 11. The final two rows are the total ionic portion of the dielectric response per direction (*x*, *y*, *z*) per mode μ (denoted as $\epsilon_{\mu }$), and the directionally decomposed electronic contribution of the dielectric response $\epsilon_{\infty }$.

System	ω	*x*	*y*	*z*
2(SA)·BPEth	38.5	0.00	-	0.05
	44.5	-	0.01	-
	56.0	-	0.01	-
	77.7 *	0.09	-	0.05
	79.9 *	-	0.15	-
	89.8 *	0.22	-	0.06
	91.3	-	0.08	-
	93.2 *	0.02	-	0.10
	96.6 *	0.12	-	0.01
	98.9	-	0.01	-
$\epsilon_{\mu }$		0.99	1.57	0.85
$\epsilon_{\infty }$		3.02	3.19	2.71

**Table 9:** Same description as Table 8, but for 2(SA)·BPE polymorphs I (top) and II (bottom) using DFT-D. Asterisks are next to the frequencies that have high contributions to $\epsilon_{\mu }$ and are plotted in Figure 12 and Figure 13 for 2(SA)·BPE polymorphs I and II, respectively.

2(SA)·BPE-I	28.9 *	0.00	-	0.08
	33.9 *	0.01	-	0.12
	34.9 *	-	0.15	-
	48.5	-	0.01	-
	50.9	0.01	-	0.02
	51.1	-	0.01	-
	61.5	0.02	-	0.02
	69.8 *	-	0.13	-
	70.3 *	0.06	-	0.02
	87.8	0.01	-	0.03
	109.5	0.06	-	0.02
$\epsilon_{\mu }$		0.58	2.19	1.66
$\epsilon_{\infty }$		2.86	3.16	3.14
2(SA)·BPE-II	41.5	-	0.01	-
	42.3 *	0.02	-	0.28
	47.3	-	0.04	-
	68.5 *	0.00	-	0.11
	69.1 *	-	0.19	-
	88.4	0.07	-	0.03
	90.9	-	0.02	-
	92.3 *	0.19	-	0.02
	102.9 *	0.24	-	0.02
	103.9	-	0.04	-
	110.2	-	0.02	-
$\epsilon_{\mu }$		0.79	1.01	0.64
$\epsilon_{\infty }$		3.44	3.72	2.83

**Table 10:** Experimentally determined ϵ of co-crystals compared to the isotropically averaged ϵ computed using DFT-D. Percent deviation from experiment is in parentheses.

	Experiment	DFT-D
2(SA)·BPE-I	6.13 ± 0.19	4.53 (−26.10)
2(SA)·BPE-II	4.89 ± 0.05	4.14 (−15.34)
2(SA)·BPEth	7.82 ± 0.15	4.11 (−47.44)
